# Expanding the non-technical skills vocabulary of operating room nurses: a qualitative study

**DOI:** 10.1186/s12912-023-01500-9

**Published:** 2023-09-19

**Authors:** Irene Sirevåg, Ingrid Tjoflåt, Britt Sætre Hansen

**Affiliations:** 1 Faculty of Health Sciences, Department of Caring and Ethics, University of Stavanger, Postboks 8600, Stavanger, 4036 Norway; 2 Faculty of Health Sciences, Department of Quality and Health Technology, University of Stavanger, Stavanger, Norway

**Keywords:** Circulating nurse, Crew resource management, Ethics, Non-technical skills, Operating room nursing, Operating theatre nursing, Perioperative nursing, Scrub nurse

## Abstract

**Background:**

Operating room nurses have specialised technical and non-technical skills and are essential members of the surgical team. The profession’s dependency of tacit knowledge has made their non-technical skills difficult to access for researchers, thus, creating limitations in the identification of the non-technical skills of operating room nurses. Non-technical skills are categorised in the crew resource management framework, and previously, non-technical skills of operating room nurses have been identified within the scope of the framework. The purpose of this study is to explore operating room nurses’ descriptions of their practices in search for non-technical skills not included in the crew resource management framework.

**Methods:**

This study has a qualitative design. An expert panel of experienced operating room nurses (N = 96) in Norway provided qualitative descriptions of their practice in a Delphi survey. The data were analysed in an inductive thematic analysis. This study was conducted and reported in line with Standards for Reporting Qualitative Research (SRQR).

**Results:**

The inductive thematic analysis developed two themes, ‘Ethical competence’ and ‘Professional accountability’, that encompass operating room nurses’ novel descriptions of their non-technical skills. The participants take pride in having the patients’ best interest as their main objective even if this may threaten their position in the team.

**Conclusions:**

This study has identified novel non-technical skills that are not described in the crew resource management framework. These findings will contribute to the development of a new behavioural marker system for the non-technical skills of operating room nurses. This system will facilitate verbalisation of tacit knowledge and contribute to an increased knowledge about the operating room nursing profession.

## Background

Operating room (OR) nursing as a profession originates from the early days of surgery on anaesthetised patients [1]. Since then, it has developed from attending the surgeon and providing ‘household’ of the OR, to a profession with advanced specialised skills. Traditionally, OR nursing relied on historical legacy, with tacit and unspoken knowledge developed within the OR, rather than theoretical principles and evidence-based knowledge [1]. The main issue with tacit and unspoken knowledge lies in the name; it is difficult to verbalise. Tacit knowledge has been described as knowledge nurses are aware of but struggle to explain [2]. With the requirements of modern healthcare and formalised OR nursing education, all relevant skills-sets must be verbalizable. Sirevåg et al. [3] found that the use of non-technical skills (NTS) vocabulary helped the OR nurses verbalise their tacit knowledge. By using the term NTS, defined as ‘the cognitive, social and personal resource skills that complement technical skills, and contribute to safe and efficient task performance’ p.1 [4], we gain a common vocabulary for the former ‘tacit skills’ [3].

When relying on NTS developing over time, as have traditionally been the case, we accept a higher risk of adverse events until novice and student OR nurses have developed their NTS. Therefore, to ensure safe surgery independent of staff experience, systematic training and, subsequently, assessment of NTS are necessary [5]. The Crew Resource Management (CRM) coursework was initially developed for training NTS in aviation [6]. Requirements from the aviation authorities led to the development of a behavioural marker system for the assessment of pilot’s NTS [6]. Subsequently, Flin et al. have constructed a CRM framework of the main NTS categories and elements based on CRM courses and NTS lists from various organisations (Table 1, Columns 1 and 2) [4]. Several behavioural marker systems for individual skills training and assessment have been developed based on the CRM system. According to Flin et al. [4], these tools must be customised with user specific behavioural markers, and the developers of such tools must define the context and identify all relevant NTS of the target users.

OR nursing is practiced by scrub and circulating nurses behind closed doors. The lack of verbalisation of their competence results in hospital administration, and even other members of the surgical team, being unaware of their full competencies and responsibilities [1, 7]. These issues may have contributed to ‘outsiders’ defining OR nursing in relation to the surgeons [7]. When examining the development of an existing NTS assessment tool, the Scrub Practitioners’ List of Intraoperative Non-Technical Skills (SPLINTS) [8], we detect that some limitations were made which may decrease the usability of the instrument. Firstly, in SPLINTS, the intraoperative timespan is defined as ‘knife-to-skin to close’ (p. 826) [9], which corresponds with the period the surgeon is present. However, the OR nursing profession defines the intraoperative timespan as the time from when the patient arrives at the OR until they are transferred to the recovery unit after surgery [10]. Furthermore, the development of the SPLINTS instrument was based exclusively on the scrub nurse’s skills, thus dismissing the NTS of the circulating nursing role [11].

The first step in developing a customised taxonomy of skills for OR nursing is to identify the nurses’ skills and behaviours that are considered to influence their safe performance [4]. Literature searches in the CINAHL and Medline databases (operating room nurs*/perioperative nurs* and non-technical skills/nontechnical skills) identified two reviews and seven empirical studies, where the NTS of OR nurses were identified to some degree with OR nurses as informants/participants. The most frequently identified NTS for scrub and circulating nurses were in the ‘communication’, ‘situation awareness’, and ‘teamwork’ categories [12–18]. Two studies identified the ‘task management’ category for scrub and circulating nurses [13, 15]. However, the ‘decision making’ and ‘leadership’ categories were only identified for circulating nurses [13, 15, 16]. Two studies found that ethical aspects influence OR nurses’ NTS [18, 19]. The research shows that the NTS of scrub nurses are more widely explored than those of circulating nurses [13–17]. However, the circulating nurses’ NTS are more advanced and autonomous than those of scrub nurses [13, 15, 16]. While most studies on NTS are based on the CRM framework; Hanssen et al. [19] shows that there may be other aspects of NTS not yet captured by the literature.

To improve the education, and promote life-long learning, regarding NTS for OR nurses we want to construct a new behavioural marker tool for NTS observation and assessment that includes the NTS of both the circulating and the scrub OR nursing role during the entire intra-operative timespan. To generate an item pool of NTS descriptions for our tool development we must include the identified NTS of scrub and circulating nurses and further explore whether there are NTS outside the CRM framework which should be included to create a customised tool.

This study aims to explore OR nurses’ descriptions of their practices in search for NTS not covered by the CRM framework.

## Methods

### Design

We have used an exploratory design with an inductive thematic analysis to explores the dimensions of NTS [20]. The Delphi technique is considered suitable for capturing collective knowledge within a group, and for researching areas that lack empirical data [21, 22] Thus, the Delphi technique was chosen to open the ‘closed doors’ of OR nursing and exploring its NTS. Although a three-round Delphi technique was conducted, the data for this study is limited to the qualitative elaborations of the second round. This study was conducted and reported in line with Standards for Reporting Qualitative Research (SRQR).

### Population and sample

The success of the Delphi technique depends upon an ‘expert’ panel [23]; thus, we defined eligible experts as being OR nurses with a minimum of 2 years post-training experience. In Norway, the qualifications required for OR nurses include being registered nurses and to have completed additional post graduate education (18 months) or Masters’ degree (24 months) specialising in OR nursing where the main difference between the educations are the writing of a master’s thesis. Furthermore, the OR nurses alternate between the scrub and circulating role throughout the day. The Norwegian Association of Operating Room Nurses distributed our invitations containing survey links to 1640 members, and we performed the convenience sampling through self-recruitment.

The inclusion criteria were:

1. OR nurse in active duty.
2. Minimum 2-years post-training experience.
3. Completion of previous Delphi round.

Scholars have discussed the proper sample size for Delphi studies in the methodological literature. For example, Keeney et al. [23] suggests balancing the ability to generate conclusions with practical management of the panel size. Therefore, we considered a panel size of approximately 100 participants to be sufficient yet feasible, and 106 participants were recruited. We expected some attrition to occur between rounds; hence, 96 OR nurses participated in the second round.

### Data collection

The Delphi survey was conducted between May and August 2020. We created and implemented the surveys in Norwegian using online survey software (SurveyXact 12.9; Ramboll Management Consulting, Copenhagen, Denmark) based on the categories and elements presented in the CRM framework [4] (Table 1). The Delphi survey was pre-tested by three fellow academics before it was piloted by 10 OR nurses meeting our inclusion criteria. A full description of the surveys for all three rounds is presented in a separate article [16]. Based on the first-round analysis we realised there were some misinterpretations of the elements; thus, we constructed the qualitative questions to operationalise and adapt the contents to the perioperative context (Table 1). In order to maintain a reasonable completion time in the second-round survey, the participants were asked to select the three most important categories of NTS. Subsequently they received follow-up questions about the selected categories and their respective elements. The panel were encouraged to write freely without limitations regarding word-count or content. We collected approximately 32,500 words of rich content from 490 text field answers. Table 1 presents the distribution of the answers.

**Table 1:** The CRM framework (Flin et al. ,2008) and corresponding survey questions with response rate

*CRM Category*	*CRM element*	*Optional Survey question, operationalised and adapted to population*	*n (%)* ^†^
*Situation awareness* *n = 56*	Gathering information	Please give examples of how you gather information (what you see, hear, smell, feel) in the OR.	35 (63)
	Interpreting information	Please give examples of how you interpret your surroundings in the OR	29 (52)
	Anticipating future states	Please give examples of how you anticipate situations, and their outcomes.	36 (64)
*Decision-* *making* *n = 26*	Defining problems	Please give examples of situations where you have encountered well-defined or poorly defined problems.	20 (77)
	Considering options	Please give examples of situations where you needed to find solutions to a problem.	20 (77)
	Selecting and implementing option	Please give examples of situations where you used your knowledge and experience to solve problems.	19 (73)
	Outcome review	Please give examples of situations where you have evaluated and reviewed your solutions.	18 (69)
*Communi-cation* *n = 60*	Sending information clearly and concisely	Please give examples of situations where you have experienced good or poor communication.	50 (83)
	Including context and intent	Please give examples that illustrates the importance of precise communication.	37 (61)
	Receiving information	Please give examples of situations where misunderstandings have occurred.	29 (48)
	Identifying and addressing barriers to communication	Please give examples of situations where you have encountered barriers for effective information.	39 (65)
*Team * *working* *n = 83*	Supporting others	How do you support other team-members? Are you supported? How does it feel to be supported/not supported?	58 (70)
	Solving conflicts	No question given	na
	Exchanging information	How does the team function when there is good or poor exchange of information?	57 (69)
	Co-ordinating activities	How does the OR nurse coordinate activities in the team?	59 (71)
*Leadership* *n = 34*	Using authority	Please give examples of situations where you have used authority to make decisions on behalf of the team, or for part of the team.	27 (79)
	Maintaining standards	Please give examples of situations where you maintain standards.	23 (68)
	Planning and prioritising	Please give examples of how you plan and prioritise in your work.	27 (79)
	Managing workload and resources	Please give examples of how you manage the workload and resources in you work.	24 (71)
*Managing* *stress* *n = 13*	*Added question*	What kind of situations have the potential to cause stress reactions in OR nurses?	8 (62)
	Identifying symptoms of stress	Please give examples of symptoms or effects of stress you have experienced or observed in team-members.	7 (54)
	Recognising effects of stress		
	Implementing coping strategies	How do you cope with stress?	7 (54)
*Coping with * *fatigue* *n = 0*	Identifying symptoms of fatigue	Please give examples of situations where you have identified signs/consequences of fatigue.	0 (0)
	Recognising effects of fatigue		
	Implementing coping strategies	Please give examples of situations where you have employed good or bad strategies for coping with fatigue.	0 (0)
†: number of respondents (percentage of eligible respondents)			

### Data analysis

**Fig. 1:**
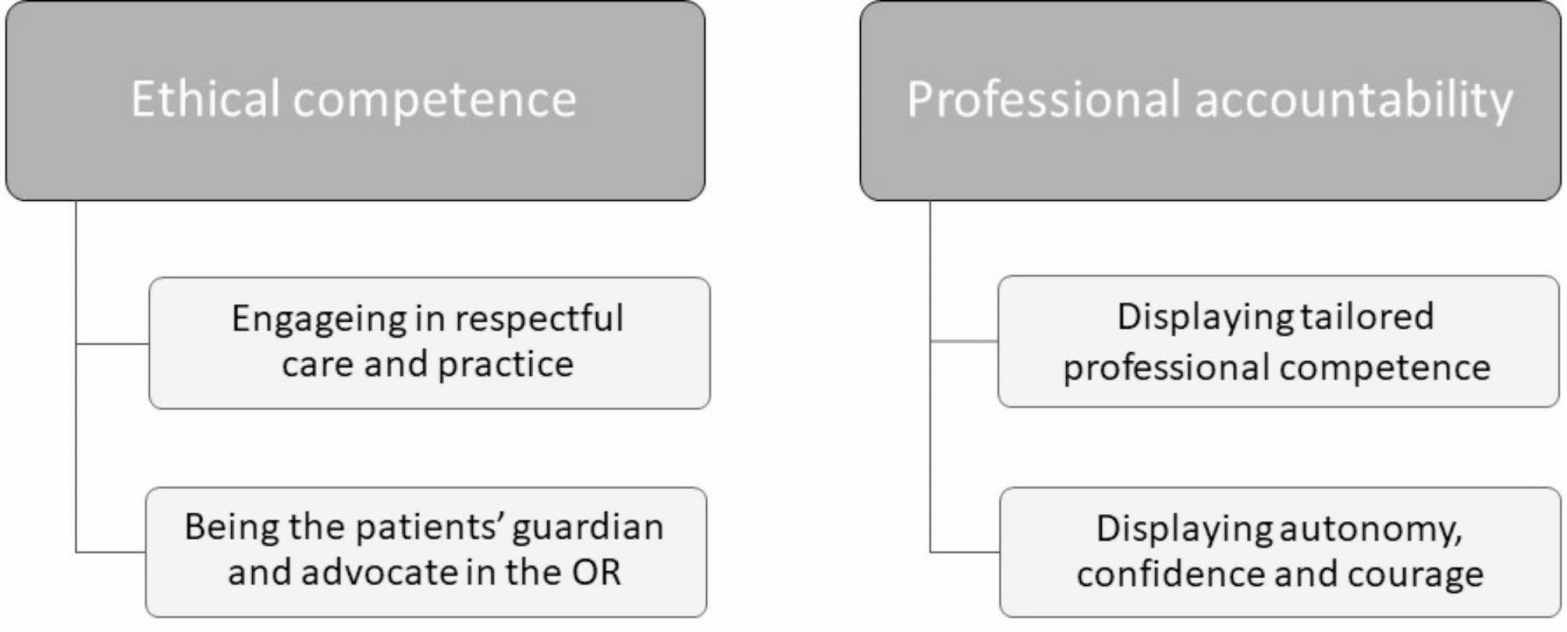
Thematic map: Themes and sub-themes

Following the initial deductive (theory-driven, CRM framework) analysis, the selective coding resulted in a large amount of uncoded, content rich data. For this study, these residual qualitative data were analysed (all authors) using inductive thematic analysis to explore the NTS in OR nursing not covered by the CRM framework [24]. The open questions led to lengthy answers and rich data, which allowed an inductive (data-driven) approach in the analyses. As the data was written, the initial transcription step recommended by Braun and Clarke was redundant [24]. The contents of all text fields were exported from SurveyXact into Microsoft Word (Microsoft, Redmond, WA, USA), and were thoroughly read and controlled for their relevance and sensitive contents. The edited Norwegian text was imported to Nvivo 12 Pro (Alfasoft, Göteborg, Sweden) where we re-read the text and annotated areas of particular interest. We searched for descriptions of OR nursing practice which were in line with the previously mentioned definition of NTS and performed a complete coding. We then revised the codes, merging similar codes. We searched for patterns across the codes and developed candidate sub-themes containing NTS descriptions. The candidate sub-themes were then reviewed, and some were merged. Sub-themes with similar contents were grouped into themes. We reviewed the codes belonging to each theme and ensured that all theme names represent their contents. Illustrative citations were translated into English by the first author. We developed a thematic map (Fig. 1) and finalised our analysis in writing up the results and discussing the findings [24]. The inductive thematic analysis developed two themes answering our aim. Thus, the OR nurses’ NTS outside CRM are organised into the themes ‘Ethical competence’ and ‘Professional accountability’.

### Ethical considerations

We conducted this research study according to the ethical principles of the Declaration of Helsinki and the Norwegian National Research Legislation [25, 26]. The study was approved by the Norwegian Centre for Research Data (ref.#: 155,726). All participants were asked to read the provided information prior to confirming their informed consent and activating the survey. The participants could withdraw at any time without ramifications. Anonymity was ensured between the panel members. However, the iteration process of the Delphi technique hinders the anonymity between researchers and participants. We treated e-mail addresses as personal information and stored them accordingly. We removed any identifying characteristics from the submitted text before analysis to provide confidentiality in the absence of anonymity [20]. All data storage protocols adhered to the requirements of the Norwegian Centre for Research Data.

### Researcher characteristics and reflexivity

Two of the authors (IS and IT) are experienced OR nurses, which provided some preconceptions during the planning of the research and the construction of the Delphi survey. Our prior understanding of the OR nursing profession, and its areas of responsibility, provided insights and facilitated a deeper understanding of the situations described by our participants. By collecting data through a survey, we did not influence the participants’ contributions. We took great care not to insert our own experiences into the data.

### Rigour

We develop the trustworthiness of the qualitative aspects of the Delphi study by demonstrating the credibility, dependability, confirmability, transferability, and authenticity of our findings according to Lincoln and Guba’s framework [27, 28]. We enhanced the credibility of the sub-study’s results by providing transparency in our expert panel selection and provided the reader with the survey questions in relation to the CRM framework. Two of the authors (IS and IT) are OR nurses and are familiar with the ‘tribal’ language in the OR. This familiarity should establish confidence in the truth of the data and their interpretations [20]. The dependability of our data refers to its stability over time and different conditions [20]. The demographic data confirm the representativeness of the expert sample in our panel [29]. The anonymity between panel members in a Delphi approach removes group bias. In addition, the panel size should ensure that a single opinion did not overpower the group. Therefore, the study findings could be repeated if our study is to be replicated with comparable participants and context. The confirmability of our results refers to their objectivity or that the data is a good representation of the participants’ information [20]. All authors actively participated in the interpretations and analysis. We ensured that no data were invented by the authors by repeatedly revisiting the original statements. The presentation of the findings includes quotes from the participants to illustrate that the interpretations are not invented by the authors. The transferability of our results refers to the extent our findings can have applicability in other settings [20]. To aid the reader in determining the level of transferability to their context, we aimed for transparency in all study steps. The inclusion criteria are presented, and the researchers did not control the self-inclusion of the participants. The authenticity of the text is enhanced by the citations which convey the mood of the participants’ experiences [20].

### Findings

Following our exploration of the OR nurses’ narratives of their practices we developed two themes, ‘Ethical competence’ and ‘Professional accountability’, which illustrates their NTS outside the CRM framework. The findings will be presented according to themes and subthemes developed in our thematic analysis guided by Braun and Clarke (Fig. 1) [24]. Due to the nature of the data collection, we are unable to pair participant numbers with citations.

### Participants

96 OR nurses completed the second Delphi round, which provided the qualitative data for this study. The expert panel members had a mean post-training experience of 24.4 years. Norwegian hospitals conduct three shifts in 24 h, and the work schedules for more than half of the panel members included evening or night shifts. Most of our participants were employed full time (35,5 h/week for shift-workers); however, half of these participants worked more than full time. Table 2 provides the participants’ demographics in detail.

**Table 2:** Demographics. Data was mainly collected in round 1, thus N = 106 unless otherwise stated

*Geographical distribution*	South-East	West	Central	North
General population of Norway†, n (%)	2900000 (56)	1100000 (21)	700000 (14)	480000 (9)
Panel members (N = 96), n (%)	52 (54)	21 (22)	16 (17)	7 (7)
*Years of post-training experience*:	2–7 years	8–14 years	14–24 years	25 + years
n (mean), mean 24.4 years	25 (4.4)	22 (10.7)	29 (19.1)	30 (30.4)
*Gender*:	Female	Male	No answer	
Members NAORN*, n (%)	1576 (96.1)	64 (3.9)		
Participants, n (%)	100 (94.3)	5 (4.7)	1 (0.9)	
*Principal area of employment*:	Operating department	Outpatient facility	Private facility	
n (%)	83 (78.3)	17 (16.0)	6 (5.7)	
*Work schedule*:	Day only	Day and shifts	Other	
n (%)	45 (42)	57 (53.8)	4 (4)	
*Work hours, percentage of full time*:	0–49%	50–74%	75–100%	> 100%
Position, (N = 96), n (%)	3 (3)	3 (3)	90 (94)	
Actual work, n (%)	2 (2)	5 (5)	48 (45)	51 (48)

†: Population numbers retrieved from the Norwegian government, www.regjeringen.no/no/dokumenter/nou-2016-25/id2522062/sec9. *: the Norwegian Association of Operating Room Nurses

### Ethical competence

The OR nurses take pride in having the patients’ best interest as their main objective throughout their work, thus displaying their ‘Ethical competence’. This theme includes two sub-themes: ‘Engaging in respectful care and practice’ and ‘Being the patients guardian and advocate in the OR’.

### Engaging in respectful care and practice

A core function of the OR nurse is to maintain a sterile field to prevent infections, even when it may be perceived as disrespectful by the surgeon: *‘I have asked the surgeon to take a break to allow me to reinforce the draping, or for the surgeon to change gloves. Often, they are not interested in taking this break, but I refuse to give them instruments until we have re-established sterility’.* The panel members consider all their actions as aspects of care, even when their patient is asleep or otherwise incapacitated. During surgery, the scrub nurses inspect every instrument for damage or residue, and they segregate all instruments contaminated by bowel, abscesses, or cysts from other instruments. All these aspects of managing asepticism illustrates how the OR nurse cares for patients by preventing surgical site infections.

Another aspect of respectful care is thoughtful positioning of patients on the operating table. Nurses mainly perform this when the patient has no, or reduced, awareness of their body or surroundings. However, the OR nurses are conscious of positioning the patient with care to prevent nerve damage, decubitus, and malignant hypothermia: ‘*As the circulating nurse, I monitor the positioning of the patient throughout the surgery. Is everything OK? Maybe their leg has slipped off the table?*’

As experienced team workers, the OR nurses recognise that each profession has their own responsibilities and that respectful cooperation is needed to reach the goal of successful surgery for their patients: ‘*This cooperation is amazing, and the joy of the work rubs off onto the patient’.* The expert panel agreed that cooperation strengthens the surgical team. However, this requires effort from all team members. Some panel members experienced a lack of understanding and respect of their work, and that they were not allowed enough time to perform their job according to their professional standards: *‘I have experienced that a patient tried to free herself from the leg supports and almost fell off the operating table […] and it was difficult to keep her safe. This happened because the anaesthesia was terminated before I had secured the patient’.* A well-functioning team depends on respect between team members. Some of the OR nurses showed their respect by *‘lifting others up’* through providing support and positive feedback.

### Being the patients’ guardian and advocate in the OR

When engaging with the patients prior to anaesthesia, the OR nurse safeguards that all the information is correct and that the patient understands what will happen to them. This creates an opportunity for discovering and rectifying issues like wrong surgical site or procedure: *’The patient was scheduled with amputation … Luckily, I knew them from their actual amputation earlier that week and knew that the correct plan for today was wound revision and change of vacuum bandages. The surgeon had copied the previous requisition.’* Panel members also described advocating for their patients during surgery: ‘*I have urged the surgeon to administer more local anaesthetics during surgery on an awake patient with unsatisfactory pain relief. The surgeon was reluctant because they were focusing on the procedure. Sometimes, when I advocate for my patient, no one listens…*’. Hence, OR nurses serve as guardians and advocates for the patient before and during their surgical procedure.

### Professional accountability

The panel members note that they have the competence to organise and manage individual procedures as well as surgical schedules. Furthermore, they must maintain their awareness of the patient, technical equipment, procedures, and the coordination needed to ensure that the patient receives safe and efficient treatment. With this professional accountability, the OR nurse contributes to minimising the risk of adverse events. Two sub-themes, ‘Displaying tailored professional competence’ and ‘Displaying autonomy, confidence, and courage’, comprise this theme.

### Displaying tailored professional competence

The panel members value their competence as highly educated health-care practitioners. They use their advanced knowledge to decide which OR is appropriate, which operating table and equipment meets the requirement for the patient’s safe positioning, and what medical or technical equipment is compatible with the surgeon’s needs and the patient’s conditions. Through extensive experience, they develop the competence to consider the consequences of their choices for the patient and the health-care personnel. Although our panel comprised experienced OR nurses, they are aware of their responsibility in training new colleagues. The novice OR nurses build competence by being supported by their experienced colleagues: *‘To see them, listen to them, and give them advice when needed. Learning is promoted when they feel safe and have a sense of coping, instead of feeling anxious’.*

The panel members’ tailored competence is also visible in their interactions with other team members. The Safe Surgery checklist is implemented in Norway [30]. However, some panel members have experienced that team members do not pay attention during ‘time-outs’ because of their simultaneous work or parallel conversations. They may miss essential information, which results in the lack of shared understanding, repetition of information, and prolonged anaesthesia time. A panel member shared an extreme consequence of not having a proper ‘time-out’: ‘*I have taken part in doing the wrong surgery on a patient after a poorly executed ‘time out’*’. Several panel members have experienced that some surgeons rush out when surgery is completed, leaving the rest of the team responsible for completing the ‘after surgery’ part of the checklist. The absence of the surgeon’s point of view may compromise the accuracy of the information passed on during the patient’s transfer into postoperative care.

### Displaying autonomy, confidence, and courage

The OR nurses position themselves as autonomous members of the surgical team who act independently rather than just following task lists and answering their colleagues’ demands: ‘*We decide when to move the patient into the OR. We decide when to call the surgeon. We decide which infection control routines to implement. We decide how to arrange the equipment. […] The OR nurses have a wide range of responsibilities’.* The panel members describe that they regularly take responsibility on behalf of others: *‘I once refused to give the cardiac surgeon his sutures because a gauze was missing. The surgeon was grumpy, but eventually he found a gauze that was used for cooling behind the heart. He thanked me later’.* One OR nurse described the frustration of having to police their colleagues and the consequences it had on a personal level: ‘*I am fed up with surgeons not adhering to the standards of preoperative hand hygiene and masks, but I am even more fed up with fellow OR nurses who don’t make sure the team follows the rules. They become more popular than me because I want to keep up my professional standards and follow the rules’.* This illustrates how the OR nurses use their professional confidence and personal courage to do the right thing for their patient, despite the hierarchical authority within the OR and the potential risk of being disfavoured by the surgeon.

## Discussion

Our findings show that the International Council of Nurses’ code of ethics are embedded in the participants’ performance. Their respect for the first code: ‘Nurses’ primary professional responsibility is to people requiring nursing care…’ [31] is illustrated by participants repeatedly stating that ‘the patient always comes first’ even when it damages their own position in the surgical team. Norms such as ethical competence and professional accountability has not previously been discussed in relation to CRM based behavioural marker tools, and it might be timely to raise the question why such rich norms for core skills are not captured by existing frameworks and tools.

### Ethical competence as non-technical skills

From an outsider’s perspective, the technical expertise of OR nurses is often perceived as the antithesis of caring with nursing and technology representing two opposing paradigms [32]. However, from within the closed doors of the OR, it is apparent that being technologically competent is perceived as being caring. Our findings illustrate that nursing in a technological environment requires the interweaving of caring and technology [33], and the tension between nursing and technology described by Barnard and Sandelowski [34] is not recognised in our data. Furthermore, while Locsin [35] theorise over the relationship between technological competency and caring, our participants considers correct handling of technical equipment and smooth instrumentation when serving the surgeon as acts of caring, which is similar to the findings of Bull and FitzGerald [33]. Intraoperative nursing care is characterised by constantly being present throughout the surgery and personalising nursing care procedures for each patient [10]. The participating OR nurses emphasised the importance of establishing a connection with their patients. However, the level of connection varies according to time allowances, with ample time given during preparation for elective surgery but only time for eye contact and maybe a reassuring touch during acute situations. In some instances, the patient may already be anaesthetised upon arrival in the OR; however, the OR nurses still care for these patients through their prevention of positioning-related injuries, surgical site infections, malignant hypothermia, or other undesired outcomes. The OR nurse’s care is mostly invisible to the patient. However, similarly to Bull and FitzGerald [33], our findings show that the OR nurses took pride in providing excellent patient care even when the patient is unaware. Thus, while Nordström & Wihlborg [36] highlight the OR nurses’ advocacy for awake patients, our study, along with Levesque et al. [37], found that the advocacy continues when the patients are unable to advocate for themselves.

Our findings illustrate that value conflicts are created between requirements for efficiency and the desire to do the best for the patients. According to Blomberg et al. [38], these value conflicts are particularly common when the surgical team do not consider the OR nurse to be competent. In these situations, our participants portrayed moral courage in their efforts to protect their patients, even if they end up being perceived as bossy or difficult by the surgical team. Blomberg et al. [38] described this moral courage as acting according to one’s conviction despite criticism from others. When the OR nurse discovers threats to their patient’s safety, such as wrongly marked surgical sites or missing surgical objects, they consult with the surgeon to rectify the issue. Therefore, they contribute to a shared risk awareness among the surgical team [39]. Such incidents may seem minor, but if not ameliorated, they may lead to major harm for the patients. Few researchers have explored the OR nurses’ error-preventing ability; however, Yang et al. [40] found that the circulating nurse play a significant role in identifying and addressing potential harmful incidents. When our panel members make decisions, they have the patients’ best interests as their guidance. Therefore, ethical considerations regarding the patients influence the OR nurses’ NTS. Few previous studies have identified the ethical aspects of NTS [18, 19]. However, Kelvered et al. [10] also found that OR nurses portrayed their ethical views and moral approach through their desire to promote their patients’ well-being. According to their descriptions of their work, the panel members position themselves as safeguards and advocates in the OR, or as Voight [41] commented, they are ‘the last line of defence for patient safety’ (p. 822).

### Professional accountability as non-technical skills

All surgical team members are experts with specialised skills that are inaccessible to someone without their training and experience. They are members of established professions that declare their responsibility for certain tasks [42]. There is some degree of division of labour within the team, which requires the knowledge of other team members’ competence and trust in their abilities [42]. Traditionally, OR nurses were trained in situ while working in the OR [1]. Following an increase in educational level, OR nurses now have more autonomous functions. However, they still have an underdeveloped language for verbalising their competence to the surgical team. Thus, OR nursing can be described similarly to an iceberg, where only the ‘above-water’ work is visible to the other professions in the perioperative team while their ‘under-water’ work is unverbalised. The lack of verbalisation results in OR nursing competence being invisible to the surgical team and also hospital administration, which is comparable to ‘the invisible work of nurses’ described by Allen [43]. This may explain why several study participants experience that their competence is underestimated, and the importance of their work underrated. Furthermore, they are not allowed sufficient time to perform their responsibilities according to the required standards. Both experienced and new OR nurses in other contexts have described this feeling of invisibility, being underrated, and not given enough time to provide quality care [36, 37, 44]. Therefore, the pursuit of efficiency to complete surgical task may cause a spiral effect, where the efficiency may hinder the surgical team’s recognition of the OR nurses’ contribution to the team [7].

The safety of the OR environment relies on procedures and standards; however, the team members adherence to these standards varies. The participating OR nurses display a sense of responsibility on behalf of other professions when they must argue with other team members to convince them to meet the given standards. Similarly, Nordström et al. [36] found that OR nurses and nurse anaesthetists took responsibility to remind other team members to do their tasks, while Flin et al. [11] underestimates the autonomy of the OR nurses and reduces their ability to ‘adhering to codes of good practice and guidelines’ (p.11). The OR nurses’ sense of responsibility on behalf of others originates from a professional obligation to keep the patient safe. However, if the corrected team members perceive this as a disturbance, the correction comes at a professional cost for OR nurses.

Successful treatments depend on the establishment of a shared understanding among the surgical team. However, the communication among team members before the patient arrives and the suspension of activities during surgical time-outs are the two most frequently missed nursing care areas in the OR [45]. Our results show that gathering the teams’ attention during time-outs is challenging. Neuhaus et al. [39] noted that the time-outs are considered burdensome by surgeons and anaesthetists. In addition, time-outs are often combined with tasks like scrubbing and draping. However, Freundlich et al. [46] found fewer disruptions and mainly full-team attendance during time-out when they were initiated by the circulating OR nurse. Levesque et al. [37] found that although the circulating nurse is in a good position to lead the surgical team in some situations, they did not receive the organisational support to lead.

A core responsibility for OR nurses is to establish and maintain an aseptic field; therefore, when they notify a team member of a breach of sterility, it is usually respected. However, some of our participants portrayed great courage and professional confidence when contesting their surgeons about missing gauzes or retained items during counting procedures. The extensive experience of our participants (mean, 23 years), and their high level of education, may influence this courage. Furthermore, the OR nurses become trusted members of the surgical team through building their experience, and thus become more comfortable speaking up [37].

Our results show that ethical competence and professional accountability are cognitive and personal resource NTS essential to patients’ safety. Similarly, Hanssen et al. (2020) identified respecting and caring for the patient in a rushed environment, and respect within the perioperative nursing team as ethical NTS. Previously, Agha et al. [47] have identified that such personal and professional values are required, along with technical and non-technical skills, for creating good surgical practices.

### Strengths and limitations of the work

The Delphi technique for data collection in this study provides both strengths and limitations. The Delphi survey was conducted during the initial months of the COVID-19 pandemic, which highly influenced data collection from healthcare workers. Face-to-face interviews were not an option during this period; however, the survey’s free text fields proved to be a robust substitute. We collected rich data from a larger and more representative group of OR nurses. However, we had limited opportunities for clarification and further elaboration. The online survey software enabled the inclusion of participants representing all Norwegian health regions and allowed the participants to use their preferred device for the survey and take breaks at their own convenience.

### Recommendations for further research

Considering the variety of qualifications required for OR nursing in different countries, our findings might not be representative in contexts where OR nurses have less education and experience than our panel members. We recommend further qualitative research studies to explore the NTS of OR nurses over different contexts. We will also recommend an exploration of NTS outside the CRM framework for other professions within the surgical team.

## Conclusions

The participants of this study have opened the closed doors to the OR. By exploring their narratives, we have identified that ‘Ethical competence’ and ‘Professional accountability’ are descriptors of OR nurses’ NTS that are essential for safe and efficient OR nursing without being included in the CRM framework. After more than a decade of constructing NTS behavioural marker systems for healthcare within the borders of the CRM framework, it may be timely to acknowledge that not all NTS in OR nursing, and the surgical team, can be drawn from those of the pilots. Our findings illustrate that not only behavioural marker systems, but also the CRM framework itself may need adaptation to allow for successful implementation into healthcare.

In a time with a global outcry over the lack of qualified OR nurses, and the threat of less qualified personnel taking over OR nursing responsibilities, verbalisation of the competence of the profession is paramount to inform hospital management and funding bodies of OR nurses’ contribution to safe and efficient surgical treatment. The authors are developing a new behavioural marker system for the NTS of OR nurses. By including these novel NTS, or (formerly) tacit skills, in a new behavioural marker system, the verbalisation of OR nursing skills will be facilitated.

## Data Availability

The datasets used and/or analysed during the current study are available from the corresponding author on reasonable request.

## List of abbreviations

OR: Operating Room
NTS: Non-technical skills
CRM: Crew Resource Management
SPLINTS: The Scrub Practitioners List of Interoperative Non-Technical Skills
